# A Multidimensional Evaluation of Intimacy

**DOI:** 10.1192/j.eurpsy.2022.2075

**Published:** 2022-09-01

**Authors:** M. Sears, L. Mcmahon, C. Crosby, B. Freihart, C. Meston

**Affiliations:** The University of Texas at Austin, Psychology, Austin, United States of America

**Keywords:** sex, relationship, Intimacy, well-being

## Abstract

**Introduction:**

Supportive, nurturing relationships facilitate good health, well-being, and life satisfaction. Intimacy is crucial for developing successful relationships as it strengthens bonds between partners through the exchange of personal details, love, and affection. Despite the importance of intimacy in developing strong relationships, the extant research often conflates affection, trust, and sexual acts with intimacy or only considers one aspect of an intimate relationship (i.e., physical or sexual touch) .

**Objectives:**

The current study aimed to clarify what elicits feelings of intimacy in men and women in order to develop a more nuanced conceptualization of intimacy for use in future research and clinical practice.

**Methods:**

In Study 1, women and men nominated over 2,700 items that “elicited feelings of intimacy” for them. Examples of nominations included: trust, communication, touch, attraction, and sex. Trained raters condensed duplicate items and created a final list of unique nominations for use in Study 2. Study 2 identified the factor structure of the nominated items by having a new sample of participants rate the extent each item elicited intimacy for them.

**Results:**

Data collection is ongoing but will be completed by December 2021. Results will be updated with an addendum after data analysis.

**Conclusions:**

will focus on gender differences in the factor structure of intimacy, how future research can avoid conflating this important construct with other relational aspects, and how a deeper understanding of intimacy can benefit treatment in clinical contexts and strengthen relationships more broadly.

**Disclosure:**

No significant relationships.

